# 以淀粉样肌病为主要表现的系统性轻链型淀粉样变性一例

**DOI:** 10.3760/cma.j.issn.0253-2727.2021.09.010

**Published:** 2021-09

**Authors:** 伟 张, 丽君 蒋, 燕萍 马, 慎 包, 俊敏 陈, 蓉 李, 秀鹏 冶, 玉萍 魏, 峰 智, 娟 田, 叶琼 李, 丽君 宋

**Affiliations:** 1 宁夏回族自治区人民医院血液内科，银川 750021 Department of Hematology, People's Hospital of Ningxia Hui Autonomous Region, Yinchuan 750021, China; 2 宁夏回族自治区人民医院宁夏老年医学中心，银川 750021 Ningxia Geriatric Center, People's Hospital of Ningxia Hui Autonomous Region, Yinchuang 750021, China

患者，男，47岁，主因“四肢无力9个月，伴四肢肿胀、僵硬、行走困难1个月”于2020年12月13日就诊于宁夏回族自治区人民医院骨科。患者入院前9个月无明显诱因出现四肢无力，就诊于当地医院，腰椎CT示L4/5及L5/S1突出，L2/3膨出，未治疗。患者入院前1个月四肢无力加重伴肌肉进行性肿胀、僵硬，逐渐累及臀部、腰背部、腹部肌肉，四肢关节屈曲受限，蹲下起立困难，上肢无法上举，每次仅能缓慢行走20 m左右即需要休息。患者既往体健。查体：腰背部、臀部、髋部、腹壁、四肢肌肉硬实呈板状，弹性消失，未触及肌肉结节或肿物，双侧肘关节轻度外翻畸形，屈曲受限，双膝关节屈曲受限，关节活动度（伸10°，曲80°），双下肢近端肌力Ⅳ^+^级，双下肢远端及上肢肌力正常，肌张力增高，四肢深浅感觉及共济运动正常。实验室检查：血常规、尿常规、便常规、外周血淋巴细胞亚群、肌钙蛋白T、肌红蛋白、自身抗体谱、肿瘤标志物均正常；外周血细胞形态学未见异常；生化常规示：ALT、AST、胆红素、ALP正常，白蛋白31.8 g/L，肌酐、尿素氮正常，尿酸473 µmol/L，β_2_-微球蛋白正常，血钙2.63 mmol/L，IgG 6.86 g/L，IgA 0.73 g/L，IgM 0.49 g/L；N末端B型利钠肽前体265.8 pg/ml；凝血功能示：纤维蛋白原降解产物9.35 mg/L，D-二聚体0.976 mg/L，余正常；红细胞沉降率：57 mm/1 h；C反应蛋白7.8 mg/L；血清免疫固定电泳检测到IgG λ型M蛋白；血清游离轻链κ 5.70 mg/L（正常参考值3.30～19.40 mg/L），λ 236.30 mg/L（正常参考值5.71～26.30 mg/L），游离轻链差值（dFLC）230.60 mg/L，κ/λ 0.024（正常参考值0.260～1.650）；24 h尿蛋白定量164.8 mg。心脏彩超示：室间隔厚度10.1 mm，射血分数68.5％。我科会诊考虑浆细胞疾病，转入血液科进一步检查。骨髓穿刺涂片可见5％成熟浆细胞，骨髓流式细胞术检测到CD38^+^异常单克隆浆细胞2.4％。右下腹皮下脂肪活检刚果红染色阳性，提示淀粉样变性。故行右肩三角肌活检：横纹肌组织，小灶间质可见淡红染物质，刚果红染色阳性，偏正光下显示苹果绿双折射，免疫组化示：λ（+），κ（−），提示λ型淀粉样变性。骨髓FISH检查：1q21基因扩增（+），RB1（13q14）基因缺失（−），TP53基因缺失（−），IgH基因重排（−）。肌电图示：肌源性损害，明显纤颤电位和正锐波；双侧腓总神经、胫后神经、坐骨神经及正中神经运动传导波幅降低，双侧腓浅神经、正中神经感觉传导减慢、波幅降低。MRI全身弥漫加权成像示：未见明显骨质破坏征象；广泛肌间隙水肿。双侧腕关节MRI示：鞘膜增厚、水肿，腕管容积减小，正中神经受压。心脏MRI钆延迟增强扫描示心内膜不均匀增厚并延迟强化。综合分析诊断为系统性轻链型淀粉样变性（以骨骼肌受累为主），根据2012梅奥标准分期为Ⅱ期，给予PD方案（硼替佐米1.3 mg/m^2^，第1、4、8、11天；地塞米松40 mg/d，第1～4天）治疗，2个疗程后患者腰背部及四肢肌肉明显变软，乏力减轻，每次可自行步行200 m左右，复查血清游离轻链κ 11.39 mg/L，λ 143.37 mg/L，dFLC 131.98 mg/L，κ/λ 0.079，目前患者仍在持续治疗中。

讨论：淀粉样肌病为系统性轻链型淀粉样变性的罕见表现形式，临床表现以骨骼肌损害为主，极易被误诊、漏诊，肌活检是确诊的关键，肌电图和骨骼肌MRI具有一定的特点但缺乏特异性、治疗采用系统性轻链型淀粉样变性的治疗策略。

